# Robotic surgery contributes to the preservation of bowel and urinary function after total mesorectal excision: comparisons with transanal and conventional laparoscopic surgery

**DOI:** 10.1186/s12893-022-01596-x

**Published:** 2022-04-21

**Authors:** Takuya Miura, Yoshiyuki Sakamoto, Hajime Morohashi, Akiko Suto, Shunsuke Kubota, Aika Ichisawa, Daisuke Kuwata, Takahiro Yamada, Hiroaki Tamba, Shuntaro Matsumoto, Kenichi Hakamada

**Affiliations:** Department of Gastroenterological Surgery, Hirosaki University Graduate School of Medicine, Zaifu-cho 5, Hirosaki, 036-8562 Japan

**Keywords:** Lower rectal cancer, Bowel function, Urinary function, Robotic, Laparoscopic, Transanal TME

## Abstract

**Background:**

Determine whether robotic surgery is more effective than transanal and conventional laparoscopic surgery in preserving bowel and urinary function after total mesorectal excision (TME).

**Methods:**

Of 79 lower rectal cancer patients who underwent function-preserving TME between 2016 and 2020, 64 patients consented to a prospective questionnaire-based functional observation study (52 responded). At 6 months post-resection or ileostomy closure, Wexner, low anterior resection syndrome (LARS), modified fecal incontinence quality of life, and international prostate symptom scores were used to evaluate bowel and urinary function, comparing robotic surgery (RTME) with transanal (taTME) or conventional laparoscopic surgery (LTME).

**Results:**

RTME was performed in 35 patients (54.7%), taTME in 15 (23.4%), and LTME in 14 (21.9%). While preoperative bowel/urinary functions were similar in all three procedures, and the distance from the anal verge to tumor was almost the same, more hand-sewn anastomoses were performed and the anastomotic height from the anal verge was shorter in taTME than RTME. At 2 years post-resection, 8 patients (12.5%) had a permanent stoma; RTME showed a significantly lower rate of permanent stoma than taTME (2.9% vs. 40%, p < 0.01). Despite no significant difference, all bowel function assessments were better in RTME than in taTME or LTME. Major LARS was observed in all taTME and LTME cases, but only 78.8% of RTME. No clear difference arose between RTME and taTME in urinary function; urinary dysfunction was more severe in LTME than RTME (36.4% vs. 6.1%, p = 0.02).

**Conclusions:**

In function-preserving TME for lower rectal cancer, robotic surgery was suggested to be more effective than transanal and conventional laparoscopic surgery in terms of bowel and urinary functions.

## Background

Bowel and urinary dysfunction affecting quality of life after function-preserving rectal resection occurs in about half of all patients, especially after total mesorectal excision (TME) for lower rectal cancer [1, 2]. Therefore, it has been expected that the high quality of surgery brought about by the effects of magnification using the endoscope would improve the functional results as well as the oncological results. In terms of oncological outcomes, the results of several randomized controlled trials have shown that conventional laparoscopic surgery is equivalent to a laparotomy, while others have shown that laparoscopic surgery is inferior [3, 4]. On the other hand, in terms of function, it has been reported that urinary function is equivalent, but bowel function may be better with conventional laparoscopic surgery than with open surgery [5, 6]. One of the reasons why laparoscopic surgery, with its superior visibility, has not been shown to be more useful oncologically and/or functionally than laparotomy is thought to be that it requires a high level of skill and that it is difficult to perform reproducible, high quality surgery due to the limitation of motion with the linear tools used in the conventional transabdominal laparoscopic approach to deep pelvic manipulation, which is directly related to the functional and oncological outcomes.

Therefore, transanal TME (taTME), which is an alternative approach to the deep pelvic region, continues to develop and has been reported to improve oncological outcomes and reduce urinary dysfunction by reliably preserving the neurovascular bundle [7, 8]. However, it is not clear whether it contributes to the reduction of bowel dysfunction; conversely, it has been suggested that it may even exacerbate it [8–10]. Another approach, using robots with features such as improved articulation that can rotate more than a human joint, shake stabilization, and stable 3D cameras, is also expected to improve the quality of surgery. Although the benefit of robotic surgery on oncological outcomes compared with open surgery has been reported [11], the oncological benefits compared with laparoscopic surgery have not been clarified [12, 13]. In addition, the usefulness of robotic surgery for preserving urinary function compared with laparoscopic surgery has been reported [14–16], but there appears to be no difference in bowel function [12, 13]; and there is no study comparing robotic surgery with taTME. For these reasons, we conducted a prospective, single-center, observational study to evaluate the efficacy of robotic surgery in preserving bowel and urinary functions, comparing conventional laparoscopy and taTME.

## Methods

### Patients

The inclusion criteria were: patients with lower rectal cancer, regardless of stage, who, on admission, were evaluated able to achieve a curative resection, were deemed eligible for function-preserving TME, and who agreed to participate in the study. The exclusion criteria were: patients with decompression stoma for preoperative therapy, simultaneous colon surgery, or anticipated non-curative resection status. The endpoint was postoperative restoration of bowel and urinary function at 6 months after it becomes possible to defecate through the anus. The sample size was set so that the number of robotic surgeries was approximately double that of other surgeries. Among 79 consecutive cases of lower rectal cancer that underwent function-preserving TME at the department of gastroenterological surgery, Hirosaki University, from August 2016 to April 2020, 64 patients were included who, after being admitted for surgery, consented to this functional observation study. Reasons for exclusion were as follows: non-consent in 8 cases (5 robotic, 3 laparoscopic), stoma status for preoperative therapy in 5 cases (3 robotic, 2 laparoscopic), simultaneous colon surgery in 1 case (1 laparoscopic), and non-curative status of another cancer (1 robotic). Lower rectal cancer was defined as palpable by digital examination, with the lower margin of the tumor located below the peritoneal reflection. Preoperative chemotherapy was given to eligible patients with cStage II–III on preoperative CT and high-resolution MRI, and if consent was obtained, surgery with lateral lymph node dissection was performed approximately 6–8 weeks after the last administration (Trial No. jRCTs021180033 and jRCTs021180023). Preoperative chemoradiation was given to two patients, including one who was treated at another hospital and one who was deemed ineligible for preoperative chemotherapy and received it at our hospital. Postoperative complications were defined according to the Clavien-Dindo classification [17]. Anastomotic leakage was classified into three grades; only Grades B and C were considered as anastomotic leakage in this study, Grade A was not included [18]. This study was conducted with the approval of the Ethics Committee of our institution (2016-074).

### Operative and perioperative management

General perioperative management included fasting and mechanical pretreatment on the day before surgery, perioperative antimicrobial therapy immediately before and for 1 day after surgery, and decompression with a transanal drain for 4 days after surgery for patients who underwent anastomosis with the double stapler technique (DST).

As a general surgical approach, the inferior mesenteric artery, the left colonic artery, and the inferior mesenteric vein were dissected, followed by TME, and bilateral lateral lymph node dissection (LLND) was added when the pretreatment tumor depth was T3 or deeper, as in our previous report [19]. Usually, in laparoscopic or robotic surgery, dissection is attempted as far as possible into the anal canal with the first attempt to transect the rectum by stapler anteriorly. If this is not possible, rectal transection is performed by transanal manipulation. At this institution, the basic approach for TME has been laparoscopic since 2014. The choice of approach during the period of this study was as follows. After cadaver training and visits to advanced facilities, we introduced taTME for lower rectal cancer in August 2016 and performed simultaneous transanal and laparoscopic operations in 10 cases until March 2017 (2016-004), and then limited taTME for men in 5 cases (2017-046). From March 2017 to May 2018, lower rectal cancer was basically treated with conventional laparoscopic surgery, and robotic surgery was performed specifically in early-stage cancer (2015-103). As robotic surgery is now covered by insurance in Japan, since June 2018, TME has been performed under robotic assistance. Anastomosis was performed in end-to-end fashion, and diverting ileostomy was constructed in all cases except for DST anastomosis in women who had not received preoperative treatment. Laparoscopic surgery was performed by one surgeon (HM), while robotic surgery was performed by two surgeons (TM, HM). TaTME and transanal manipulations in laparoscopic or robotic operations were performed by one surgeon (TM). If clinical anastomotic leakage was suspected after surgery, the diagnosis was confirmed by fluoroscopy or CT scan.

### Questionnaire

Preoperative assessments of bowel and urinary function were performed by giving a questionnaire to patients with lower rectal cancer, after admission, who were judged to be eligible for function-preserving TME. Assessment of postoperative function at 6 months after resection or stoma closure was determined by a mail questionnaire. The questionnaire was designed with questions that allowed calculation of Wexner Score [20], LARS (low anterior resection syndrome) score [21], mFIQL (modified fecal incontinence quality of life) Score [22], IPSS (international prostate symptom score) [23]. Severe dysfunction was defined as Wexner score ≥ 10 (median), LARS score ≥ 30 (Major), mFIQL Score ≥ 50 (median), or IPSS ≥ 21 (Severe).

### Statistical analyses

The categorical variables were compared using Fisher’s exact test and continuous variables with the Mann–Whitney U test. The rate of permanent stoma at 2 years was compared using the Kaplan–Meier method with a log-rank test. A two-sided p < 0.05 was considered statistically significant, and all statistical analyses were performed using SPSS version 26 (IBM Inc., Armonk, NY).

## Results

### Patient characteristics

The median age was 66 years (range: 34–81), 50 (78.1%) were male, and the median body mass index was 22.8 (range: 18.6–31.4) (Table 1). There were 25 (39.1%) patients with hypertension, 11 (17.2%) patients with diabetes mellitus, and 3 (14.7%) patients with severe complications of ASA 3, 4 or more. The median tumor diameter was 34.5 mm (range: 0–80) and the median distance from the anal verge to the lower margin of the tumor was 50 mm (range: 25–80). There were 39 (60.9%) cT3 and 23 (35.9%) cN positive patients, and preoperative treatment was given to 36 (56.3%) patients. Robotic TME was performed in 35 patients (54.7%), taTME in 15 patients (23.4%), and conventional laparoscopic TME in 14 patients (21.9%). The double staple technique was used in 32 patients (50%), and the hand sewn method was used in 32 patients (50%), for all of whom end-to-end anastomosis was performed. The median anastomotic height from the anal verge was 40 mm (range: 20–60). Diverting ileostomy was created in 61 (95.3%) patients. Lateral lymph node dissection was performed in 38 patients (59.4%), 36 of whom had preoperative treatment. The median operative time was 395 min (170–639), and the median blood loss was 50 ml (0–3137). There were no surgery-related deaths, and 6 patients (9.4%) had Clavien-Dindo classification III or higher. Anastomotic leakage was observed in 5 patients (7.8%) overall, 4 patients (6.2%) with Grade B and 1 patient (1.6%) with Grade C. The pathological staging was as follows: stage 0, 11 patients (17.2%), stage I, 28 patients (43.8%), stage II, 12 patients (18.8%), stage III, 11 patients (17.2%), and stage IV, 2 patients (3.1%). A radial margin of ≤ 1 mm was found in 3 patients (4.7%).

**Table 1:** Clinicopathological characteristics

Item	Value
Age (year)^†^	66 (34–81)
Gender, n (%)	
Male	50 (78.1)
Female	14 (21.9)
Body mass index (kg/m^2^) †	22.8 (18.6–31.4)
Smoking, n (%)	22 (34.3)
Hypertension, n (%)	25 (39.1)
Diabetes, n (%)	11 (17.2)
ASA ≥ 3, n (%)	3 (4.7)
Tumor size (mm) †	34.5 (0–80)
Distance from anal verge to tumor (mm) †	50 (25–80)
cT3, n (%)	39 (60.9)
cN positive, n (%)	23 (35.9)
Preoperative treatment, n (%)	36 (56.3)
Approach, n (%)	
Robotic	35 (54.7)
Transanal	15 (23.4)
Laparoscopic	14 (21.9)
Type of anastomosis, n (%)	
Double staple technique	32 (50.0)
Hand sewn	32 (50.0)
Anastomotic height from anal verge (mm)^†^	40 (20–60)
Diverting stoma, n (%)	61 (95.3)
Lateral lymph node dissection, n (%)	38 (59.4)
Operation time (min)^†^	395 (170–639)
Blood loss (ml)^†^	50 (0–3137)
Complications (Clavien-Dindo), n (%)	
I–II	39 (56.2)
III	5 (7.8)
IV	1 (1.6)
V	0 (0)
Anastomotic leakage, n (%)	5 (7.8)
Grade B	4 (6.2)
Grade C	1 (1.6)
Pathological TNM stage, n (%)	
0	11 (17.2)
I	28 (43.8)
II	12 (18.8)
III	11 (17.2)
IV	2 (3.1)
Radial margin ≤ 1 mm, n (%)	3 (4.7)

*ASA* American Society of Anesthesiologists

^†^Median (Range)

### Comparison of clinicopathological factors, preoperative function, and permanent stoma

There were no obvious differences in clinical factors between the robotic and the taTME groups, except for age, which was significantly higher in the taTME group (Table 2). In terms of surgical factors, the taTME group had significantly more hand-sewn anastomoses, a lower anastomotic height from the anal verge, a shorter operative time, and a longer distal margin. On the other hand, there was no obvious difference in the preoperative bowel and urinary functions between them. In the comparison between the robotic and laparoscopic groups, there was no obvious difference in clinical and surgical factors, but the rate of pStage 0-I was significantly higher in the robotic group. There was also no clear difference in the preoperative bowel and urinary functions between them. At the median observation period of 30 months (range: 7–54) after resection, 8 patients (12.5%) had a permanent stoma at 2 years after resection, and the robotic group had a significantly lower rate of permanent stoma at 2 years compared to the taTME group (2.9% vs. 40%, p < 0.01). There was no difference when compared to the laparoscopic group. In the taTME group several patients ended up with permanent stoma; two had pelvic abscesses, three experienced poor bowel functions, and one never had the stoma closed, as this patient died of another disease.

**Table 2:** Clinicopathological and preoperative functional comparisons between robotic and transanal or laparoscopic approach

Variables	Robot	taTME	*P* value*	Laparoscopy	*P* value**
	N = 35	N = 15		N = 14	
Age^†^	65 (37–75)	70 (38–81)	0.03	66 (34–77)	0.65
Gender (male), n (%)	28 (80.0)	14 (93.3)	0.40	8 (57.1)	0.15
Body mass index (kg/m^2^)^†^	23.0 (18.7–31.4)	23.3 (19.1–28.9)	0.97	21.4 (18.6–28.8)	0.29
Smoking, n (%)	9 (25.7)	5 (33.3)	0.73	8 (57.1)	0.05
Hypertension, n (%)	13 (61.9)	8 (38.1)	0.35	4 (28.6)	0.74
Diabetes, n (%)	4 (11.4)	5 (33.3)	0.10	2 (14.3)	1.00
ASA ≥ 3, n (%)	2 (5.7)	0 (0)	1.00	1 (7.1)	1.00
Tumor size (mm)^†^	34 (0–80)	38 (0–75)	0.96	37 (0–57)	0.85
Distance from AV to tumor (mm)^†^	55 (25–80)	50 (30–60)	0.17	57 (35–80)	0.29
cT3, n (%)	22 (62.9)	7 (46.7)	0.35	10 (71.4)	0.74
cN positive, n (%)	14 (40.0)	2 (13.3)	0.09	7 (50.0)	0.54
Preoperative treatment, n (%)	21 (60.0)	7 (46.7)	0.53	8 (57.1)	1.00
Hand sewn, n (%)	12 (34.2)	15 (100)	< 0.01	5 (35.7)	1.00
Anastomotic height from AV (mm)^†^	40 (20–60)	25 (20–40)	< 0.01	50 (20–60)	0.52
Lateral lymph node dissection, n (%)	21 (60.0)	7 (46.7)	0.53	4 (71.4)	0.52
Operation time (min)^†^	465 (299–631)	317 (170–396)	< 0.01	430 (185–639)	0.39
Blood loss (ml)^†^	50 (0–440)	60 (5–230)	0.33	107 (0–3137)	0.91
Clavien-Dindo ≥ 3, n (%)	1 (2.9)	2 (13.3)	0.21	3 (21.4)	0.06
Anastomotic leakage, n (%)	1 (2.9)	2 (13.3)	0.21	2 (14.3)	0.19
pStage 0-I, n (%)	23 (65.7)	12 (80.0)	0.50	4 (28.6)	0.02
Distal margin (mm)^†^	15 (5–30)	20 (10–35)	0.02	20 (5–30)	0.19
Radial margin ≤ 1 mm, n (%)	0 (0)	1 (6.7)	0.30	2 (14.3)	0.07
Wexner score^†^	1 (0–16)	2 (0–4)	0.55	1 (0–18)	0.55
Wexner ≥ 10, n (%)	1 (2.9)	0 (0)	1.00	1 (7.1)	0.49
LARS score^†^	11 (0–39)	15 (0–38)	0.71	11 (0–41)	0.22
major LARS, n (%)	3 (8.6)	2 (13.3)	0.62	4 (28.6)	0.09
mFIQL score^†^	0 (0–57.1)	0 (0–23.8)	0.88	0 (0–90.4)	0.90
mFIQL ≥ 50, n (%)	2 (5.7)	0 (0)	1.00	1 (7.1)	1.00
IPSS^†^	5 (0–27)	7 (3–29)	0.06	5.5 (1–21)	0.66
Severe IPSS, n (%)	1 (2.9)	2 (13.3)	0.21	1 (7.1)	0.49
Observational periods (month)^†^	22 (9–47)	43 (7–54)	< 0.01	33 (7–52)	0.01
Permanent stoma at 2 years, n (%)	1 (2.9)	6 (40.0)	< 0.01^¶^	1 (7.1)	0.97^¶^

*ASA* American Society of Anesthesiologists, *AV* anal verge, *taTME* transanal total mesorectal excision, *LARS* low anterior resection syndrome, *mFIQL* modified fecal incontinence quality of life, *IPSS* international prostate symptom score

^†^Median (Range)

*Robotic vs Transanal; **Robotic vs Laparoscopic; ^¶^Log-rank test

### Comparison of postoperative bowel and urinary function

Of the 56 patients who received the mailed questionnaire, excluding 7 stoma-dependent patients and 1 patient who died of other diseases at 6 months after stoma closure, 3 patients (1 robotic, 2 taTME, and 1 laparoscopic) did not respond. In the end, we received responses from 52 patients, for a response rate of 92.8%. Although the robotic group did not show significantly better results than the taTME or laparoscopic groups, the robotic group tended to come out better on all bowel function assessment items (Table 3). All patients in the taTME and laparoscopic groups had major LARS, but 78.8% of patients in the robotic group had major LARS. There was no clear difference in urinary function between the robotic and taTME groups, but there was significantly more severe urinary dysfunction in the laparoscopic group than in the robotic group (6.1% vs. 36.4%, p = 0.02).

**Table 3:** Postoperative functional comparisons between robotic and transanal or laparoscopic approach

Variables	Robot	taTME	*P* value*	Laparoscopy	*P* value**
	N = 33	N = 8		N = 11	
Wexner score^†^	10 (0–20)	12 (4–17)	0.13	11 (6–19)	0.16
Wexner ≥ 10, n (%)	17 (51.5)	6 (75.0)	0.42	7 (63.6)	0.72
LARS score^†^	35 (11–41)	38 (33–42)	0.22	35 (31–41)	0.08
Major LARS, n (%)	26 (78.8)	8 (100)	0.31	11 (100)	0.16
mFIQL score^†^	38.0 (0–85.7)	53.5 (10–100)	0.13	40.4 (2.38–100)	0.43
mFIQL ≥ 50, n (%)	10 (30.3)	5 (62.5)	0.11	5 (45.5)	0.46
IPSS^†^	3 (0–23)	3 (1–16)	0.35	8 (0–29)	0.09
Severe IPSS, n (%)	2 (6.1)	0 (0)	1.00	4 (36.4)	0.02

*taTME* transanal total mesorectal excision, *LARS* low anterior resection syndrome, *mFIQL* modified fecal incontinence quality of life, *IPSS* international prostate symptom score

^†^Median (Range)

*Robotic vs Transanal; **Robotic vs Laparoscopic

When bowel function of hand-sewn anastomosis was compared with DST in the robotic group, the hand-sewn anastomosis group had significantly shorter distances from tumor to anal verge and lower anastomotic height from anal verge, and higher Wexner and LARS scores (Table 4). In the robotic group, all patients with hand-sewn anastomosis had major LARS, and only 68.2% of patients with DST had major LARS. On the other hand, there was no clear difference in bowel and urinary functions between patients with or without lateral lymph node dissection. All patients with LLND received neoadjuvant therapy, which consisted of chemotherapy in 18 patients and chemoradiotherapy in one patient. Patients without LLND received no preoperative therapy.

**Table 4:** Postoperative functional comparisons by procedures in the robotic group

Variables	DST	Hand sewn	*P* value	LLND	No LLND	*P* value
	N = 22	N = 11		N = 19	N = 14	
Distance from AV to tumor (mm)^†^	60 (40–80)	35 (25–50)	< 0.01	55 (30–80)	57.5 (25–70)	0.98
Anastomotic height from AV (mm)^†^	50 (30–60)	25 (20–30)	< 0.01	40 (20–50)	42.5 (20–60)	0.79
Wexner score^†^	7 (0–16)	12 (9–20)	< 0.01	11 (0–20)	9 (1–16)	0.88
Wexner ≥ 10, n (%)	8 (36.4)	9 (81.8)	0.02	11 (57.9)	6 (42.9)	0.49
LARS score^†^	34 (11–41)	39 (34–41)	< 0.01	36 (11–41)	34.5 (15–41)	0.34
Major LARS, n (%)	15 (68.2)	11 (100)	0.06	16 (84.2)	10 (71.4)	0.42
mFIQL score^†^	29.2 (0–73.8)	45.2 (9.5–85.7)	0.06	40.9 (2.3–85.7)	32.1 (0–73.8)	0.54
mFIQL ≥ 50, n (%)	6 (27.3)	4 (36.4)	0.69	6 (31.6)	4 (28.6)	1.00
IPSS^†^	2 (0–23)	5 (0–16)	0.49	3 (0–23)	2 (0–12)	0.08
Severe IPSS, n (%)	2 (9.1)	0 (0)	0.54	2 (10.5)	0 (0)	0.49

*AV* anal verge, *LARS* low anterior resection syndrome, *mFIQL* modified fecal incontinence quality of life, *IPSS* international prostate symptom score, *DST* double staple technique, *LLND* lateral lymph node dissection

^†^Median (Range)

## Discussion

Bowel dysfunction, which occurs in many patients after rectal surgery, includes fecal incontinence, urgency, and frequent bowel movements. The LARS score has been developed as a method of evaluation for bowel dysfunction and has been translated and validated in many languages [21, 24]. In addition, function-preserving TME for lower rectal cancer is a major factor that induces fecal incontinence [25]. It is known that fecal incontinence is associated with a unique decrease in QOL, so the fecal incontinence quality of life score (FIQL) questionnaire and the modified FIQL have been developed as evaluation methods of this phenomenon [22, 26]. On the basis of these results, it was clarified that the factors inducing bowel dysfunction after function-preserving rectal resection were low anastomosis and preoperative radiotherapy [27–29]. The modified FIQL has also been used to demonstrate the functional validity of intersphincteric rectal resection [30].

This simple assessment of function has made it possible to compare new treatment strategies and is now one of the indicators for clarifying the usefulness of laparoscopic surgery in preserving function. In this study, all patients that underwent conventional laparoscopic surgery and taTME had major LARS; however, with robotic surgery, 1 in 5 patients avoided major LARS. And the number of patients with permanent stoma was significantly reduced in robotic surgery compared to taTME. One of the reasons for this may be the reduction in short-term postoperative complications, especially anastomotic leakage, which is supported by recent reports that robotic surgery contributes to a reduction in short-term complications compared with conventional laparoscopy [31]. In addition, a stable field of view and delicate dissection in the deep pelvis were achieved in the robotic modality, which may have contributed to the preservation of fine sphincter nerves such as the internal anal sphincter nerve and the levator ani nerve [32, 33]. In addition, anterior dissection in the anal canal can be performed more easily using a robot than conventional laparoscopy, and this may reduce damage to sphincter muscles which was also estimated to occurred in taTME because of the use of transanal platform and the longer time to manipulate the anus.

LLND or preoperative radiation therapy may be added to standard TME to improve local control of locally advanced lower rectal cancer [34, 35]. In Japan, LLND has been described as a standard treatment strategy for locally advanced lower rectal cancer in the official guidelines [36]. In recent years, the necessity of LLND has been pointed out in selected cases even in Europe and the United States [37]. A study with a small number of cases reported that LLND has an adverse effect on bowel function [38]. In this study, LLND had no influence on bowel function in the robotic group. On the other hand, preoperative radiotherapy is known to be a clear factor in bowel dysfunction [39]. Under such circumstances, several clinical trials on preoperative chemotherapy are being conducted in anticipation of reaping the dual effects of improving oncological control when substituted for radiotherapy and avoiding bowel dysfunction caused by radiotherapy [40, 41]. In recent years, preoperative chemotherapy has been shown to be as effective as preoperative chemoradiation in local control and reducing bowel dysfunction, suggesting that it is a useful treatment strategy [42]. At our institution, preoperative chemotherapy for locally advanced rectal cancer has been conducted as part of a clinical study [43]. However, even with the strategy of avoiding radiation, the rate of bowel dysfunction after TME, especially after hand-sewn anastomosis, was shown to be extremely high in this study, so new treatment strategies should be developed. Currently, the results of the watch-and-wait strategy (WW) have been reported in patients with complete response after chemoradiotherapy, and its oncological safety and efficacy have been clarified [44]. In addition, WW was shown to be more effective than function-preserving rectal resection after chemoradiotherapy in terms of bowel function [45]. However, in this report, it was found that chemoradiotherapy itself may also cause bowel dysfunction, and it is unclear whether TME alone or chemoradiotherapy alone is more harmful to bowel function. In recent years, WW has been shown to be associated with milder bowel dysfunction than TME alone [46]. However, because it was a retrospective study, its interpretation is controversial due to the heterogeneity of the observation period and surgical approach. In particular, prospective comparative studies between WW and robotic DST anastomosis which is suggested to be useful in this study are warranted.

In terms of urinary function, it has been reported that robotic surgery is more effective than conventional laparoscopic surgery at both six and 12 months postoperatively [14–16]. Also in this study, robotic surgery has been shown to be more effective in preserving urinary function than conventional laparoscopic surgery. On the other hand, postoperative urinary function was comparable between taTME and robotic surgery. Compared with conventional laparoscopic surgery, taTME was also reported to be effective in preserving urinary function, and the results of this study support this [8]. In consideration of the preservation of both bowel and urinary functions, the results of this study suggest that robotic surgery may be a better approach for function-preserving TME.

There were several limitations to this study. Although this was a prospective observational study, it is possible that robotic surgery had better results reflecting experience with conventional laparoscopy and taTME. The taTME group differed from the conventional laparoscopic group with regard to the fact that the results were obtained from the very first case, which may have resulted in poor results due to the initial learning curve. In addition, since all anastomoses in the taTME group were hand-sewn in the anal canal, the results may have been different had a mechanical anastomosis been performed above the anal canal. Laparoscopic surgery was performed by a single surgeon, and the results may have differed had they been obtained from multiple surgeons, thus the study is lacking some external validity. The favorable results in the robotic group may, in part, be attributable to lower age, earlier pathologic stage, and lower rate of anastomotic leakage. Despite the existence of these limitations, we believe that one of the advantages of robotic surgery is that good results can be expected from the introduction of robotic surgery in the early stages of use, because these results are from the earliest cases of robotic surgery at the institution and from two different surgeons.

## Conclusions

In function-preserving TME for lower rectal cancer, robotic surgery was suggested to be more effective in preserving bowel and urinary functions than taTME or conventional laparoscopic surgery. From the practitioner’s perspective this is especially true in the early stage of the learning curve and had translated into optimal results from the earliest cases.

## Data Availability

The datasets used and/or analyzed during the current study are available from the corresponding author upon request.

## Abbreviations

TME: Total mesorectal excision
LARS: Low anterior resection syndrome
mFIQL: Modified fecal incontinence quality of life
IPSS: Iinternational prostate symptom score
RTME: Robotic total mesorectal excision
taTME: Transanal total mesorectal excision
LTME: Laparoscopic total mesorectal excision
DST: Double stapler technique
LLND: Lateral lymph node dissection
ASA: American Society of Anesthesiologists
AV: Anal verge
WW: Watch-and-wait strategy
